# Performance Analysis and Beamforming Design of a Secure Cooperative MISO-NOMA Network

**DOI:** 10.3390/s21124180

**Published:** 2021-06-18

**Authors:** Mujtaba Ghous, Ziaul Haq Abbas, Ahmad Kamal Hassan, Ghulam Abbas, Thar Baker, Dhiya Al-Jumeily

**Affiliations:** 1 Telecommunication and Networking (TeleCoN) Research Center, GIK Institute of Engineering Sciences and Technology, Topi 23640, Pakistan; mujtaba.ghous@giki.edu.pk; 2 Faculty of Electrical Engineering, GIK Institute of Engineering Sciences and Technology, Topi 23640, Pakistan; ziaul.h.abbas@giki.edu.pk (Z.H.A.); akhassan@giki.edu.pk (A.K.H.); 3 Faculty of Computer Sciences and Engineering, GIK Institute of Engineering Sciences and Technology, Topi 23640, Pakistan; abbasg@giki.edu.pk; 4 Department of Computer Science, University of Sharjah, Sharjah P.O. Box 27272, United Arab Emirates; 5 School of Computer Science and Mathematics, Liverpool John Moores University, Liverpool L3 3AF, UK; d.aljumeily@ljmu.ac.uk

**Keywords:** MISO-NOMA, relaying transmission, optimization, outage probability, secrecy

## Abstract

This paper studies the cell-edge user’s performance of a secure multiple-input single-output non-orthogonal multiple-access (MISO-NOMA) system under the Rayleigh fading channel in the presence of an eavesdropper. We suppose a worst-case scenario that an eavesdropper has ideal user detection ability. In particular, we suggest an optimization-based beamforming scheme with MISO-NOMA to improve the security and outage probability of a cell-edge user while maintaining the quality of service of the near-user and degrading the performance of the eavesdropper. To this end, power allocation coefficients are adjusted with the help of target data rates of both the users by utilizing a simultaneous wireless information and power transfer with time switching/power splitting protocol, where the near-user is used to forward the information to cell-edge user. The analytical results demonstrate that our beamformer analysis can achieve reduced outage probability of cell-edge user in the presence of the eavesdropper. Moreover, the provided simulation results validate our theoretical analysis and show that our approach improves the overall performance of a two-user cooperative MISO-NOMA system.

## 1. Introduction

Non-orthogonal multiple-access (NOMA) has been suggested as the emerging candidate technology to improve spectral efficiency by mapping multiple users on resource domains (e.g., frequency and time) in order to enable 5G and future generation data networks, also known as future communication networks [1]. Recently, the research community is taking great interest in the integration of wireless power transmission and NOMA communication [2]. The motivation behind this technique is its potential in the improvement of energy efficiency as well as the spectral performance in these wireless networks. The need for low latency traffic and high spectral performance has resulted from an increased convergence, and it has prompted recent advancements in wireless technology [3]. NOMA stands out for its dominance in meeting the criteria of vast convergence, effectively reducing transmission latency, and improving spectral efficiency [4]. The common resource across various power levels for all superposed transmit signals is the main characteristics of NOMA. To completely exploit the advantages of NOMA, a successive interference cancellation (SIC) detector having low complexity is used on the receiving side [5]. The user, having strong channel conditions, named as a near-user, initially decodes the message of far-user that has weaker channel conditions and then eliminates the interference from the far-user by using SIC [6]. Information of the far-user is easily detected by considering all other messages as noise, given that both the users will utilize complete resource block [7].

As both the near- and far-users coexist in any communication network, the authors of [8] suggested and evaluated a novel cooperative NOMA algorithm to increase the far-user’s reliability. The fundamental theory behind this cooperative transmitting technique is that users having higher channel gains are used as relays and they assist far-users having bad channel conditions. Furthermore, based on the available limited energy storage capability at the relay nodes, compensation usually exist between information receiving and forwarding, particularly to meet internet of things (IoT) accessibility requirements [9]. As a result, multiple attempts are recorded to deploy energy harvesting (EH) wireless networks, which provide self-sustainability and the ability to share energy among nodes. The authors of [10] investigated wireless-powered NOMA networks in which energy-constrained users collect energy from the base station for conducting NOMA transmission.

By using NOMA’s power-domain multiplexing, users must consume marginal energy to power their SIC decoders [11]. This essentially prevents NOMA from being used in energy-constrained IoT. The simultaneous wireless information and power transmission (SWIPT) has recently been lauded as a promising strategy for solving the power usage challenge in wireless networks [12]. The SWIPT has been widely investigated in previous studies, and can be split into two groups on the basis of EH model, named as a linear and a nonlinear EH model. The optimum resource allocation was developed in [13] relying on the linear EH model to fulfill various performance criteria, e.g., energy consumption, throughput, and data rate fairness among others. In [14], first nonlinear EH model was developed on the basis of data measurement to delineate the nonlinear function of the energy harvester. The nonlinear EH model was used to establish the optimal resource scheduling and interference management for cognitive SWIPT networks in [15]. In [16], the authors built on their previous work to incorporate a situation of incomplete channel state information (CSI). The problem of acquiring CSI and suppressing the direct-link interference through a cloud radio access network architecture was discussed in [17], where primary and secondary users are connected to a cloud processor. A store and transmit (SaT) scheme was proposed in [18] to perform information transmitting and energy storing in a two-user NOMA communication system by using a nonlinear EH relay.

On the other hand, there is a security drawback due to the broadcast existence of wireless networks [19]. Such as, if the eavesdropper is successful in intercepting message signal of a user in a NOMA setup, then the eavesdropper would be able to collect multiple users’ information [20]. However, in the NOMA scheme, the security problem is more challenging. Physical layer security (PLS) can provide a potential defense against a malicious user’s attack. Moreover, the message will be conveyed in confidence if the legitimate user’s channel and the eavesdropper channel can be managed in such a way that the message is not intercepted. To avoid eavesdropping attacks, PLS is a forefront security technique in wireless communication [21,22]. The PLS offers keyless knowledge authentication with information-theoretic assurances, as opposed to traditional cryptography techniques that use the phenomenon of secret key exchange at both transmitter and receiver and it also depends on the decipherer’s restricted computation capacity to safeguard the information [23]. The PLS follows the Shannon theory concept, which protects the physical layer by using the inherent randomness of wireless networks [24].

The cooperative NOMA systems with PLS have recently increased researchers’ interest in this area. The authors of [25] developed closed-form secure outage probability (SOP) expressions of cooperative NOMA systems in amplify-and-forward (AF) and decode-and-forward (DF) relay modes, demonstrating that using the optimum power allocation parameters will enhance the system’s secure performance. To make a secure communication system, jamming phenomenon in NOMA cooperative system was proposed in [26]. The cooperative NOMA’s reliability and security in downlink scenario cognitive networks was evaluated by the authors in [27]. In [28], the authors suggested a cooperative jamming technique to confuse relays and indicated that fading parameters and transmission power affect secure efficiency. For secure communication connection from eavesdropping, two-stage protected relay selection strategy at both sender and receiver using NOMA was suggested in [29] to increase the ability among users. In addition, artificial noise can be used to enhance transmitting information security. Therefore, the artificial noise technique in [30] was used to increase the secrecy efficiency of multiple antenna NOMA networks. The authors of [28] studied the secrecy outage possibility (SOP) of NOMA networks via multiple relays using PLS and cooperative communication. A framework with imperfect channel estimation was used in [31] to analyze the secrecy performance of an artificial noise-aided massive multiple-input multiple-output non-orthogonal multiple-access (MIMO-NOMA) network. In particular, the asymptotic expressions and ergodic secrecy rates for users were derived in this work. A joint power allocation scheme was used in [32] for the secrecy performance analysis of a massive MIMO-NOMA network in the presence of a multiple-antenna eavesdropper. The authors in [33] analyzed the large-scale NOMA systems in the domain of PLS and characterized the SOP using homogeneous Poisson point processes (HPPPs) to spread legitimate users (LUs) and eavesdroppers (Eves). In addition, the authors of [34] looked at the PLS of an uplink NOMA where the Eves are distributed randomly, and stochastic geometry approach was used for the performance evaluation.

Despite the fact that cooperative NOMA systems with SWIPT have been considered in the literature, no research is conducted to investigate the outage probability minimization problem in such a way to design an energy-efficient beamforming strategy in a nonlinear EH model to minimize the outage probability of far-user and also degrade the performance of Eve for a secure multiple-input single-output non-orthogonal multiple-access (MISO-NOMA) communication.

### Contributions

The future deployment criteria—energy efficiency, minimum cost, and low power—implied in the application of a MISO antenna design all motivated us to explore optimized beamforming design in cooperative MISO-NOMA with SWIPT. A general MISO-NOMA system is considered here by using the SWIPT protocol where Alice (user-A), Bob (user-B), and an eavesdropper (Eve) are deployed in such a way that user-B and Eve are at the cell-edge. The decode-and-forward SWIPT architecture with EH receiver is employed at user-A using time switching/power splitting (TS/PS) protocol. User-A plays the role of a relay to forward the message signal of user-B. To this end, a cooperative scheme, where BS and each user are equipped with *N* number of antennas and single antenna, respectively, is proposed in this paper. Furthermore, the selection combining technique is employed at user-B and Eve. The main contributions of this paper are summarized as follows.

- For the general channel model, we incorporate the beamforming at the transmitter side and use the indefinite quadratic form (IQF) approach to build a signal-to-interference-plus-noise ratio (SINR) in a canonical quadratic formulation for both direct and relaying transmissions.
- The exact closed-form outage probability expressions of the legitimate users (user-A and user-B), and the Eve are derived and validated under several design parameters.
- The analysis of beamformer is performed. More precisely, the proposed beamforming finds reduced outage probability of user-B, and further degrades the performance of Eve while maintaining quality-of-service (QoS) of user-A.

The remaining part of the paper is sectionalized as follows. In Section 2, the system model and the SINR formulation of direct and relaying transmission are presented. The optimal analysis strategy and outage probability expressions of user-A, user-B, and Eve are presented in Section 3. The system model is validated in Section 4 through simulation results, while Section 5 concludes the paper.

## 2. System Model

**Figure 1:**
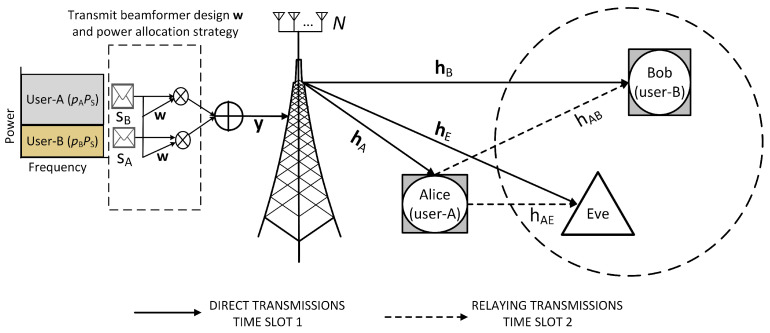
System model of a secure cooperative multiple-input single-output non-orthogonal multiple-access (MISO-NOMA) system.

We consider a downlink MISO-NOMA system consisting of one near-user i.e., user-A, one far-user i.e., user-B, a passive Eve, and a BS consisting of *N* antennas, as shown in Figure 1. Let $h_{A}$, $h_{B}$, and $h_{E}$ denote the channel vectors from BS to user-A, user-B, and Eve, respectively. Similarly, the channels from user-A to user-B, and user-A to Eve are denoted by $h_{\mathrm{AB}}$ and $h_{AE}$, respectively. Rayleigh flat fading is assumed in all wireless channels and considered as Gaussian random variables having zero mean. Additionally, $n_{A}\left(n\right)$ and $n_{B}\left(n\right)$ represent the additive white Gaussian noise (AWGN) with zero mean at user-A and user-B, respectively, having variance $\sigma_{a}^{2}$ and $\sigma_{c}^{2}$. Furthermore, the channel gain is written as $E[|h_{\mathrm{AB}}{|}^{2}]=\frac{\beta }{{\left(d_{xy}/d_{o}\right)}^{\epsilon }}$, where β is the power attenuation, ϵ denotes the path loss exponent, $d_{xy}$ is the distance between two nodes, and $d_{o}$ is the reference distance.

**Figure 2:**
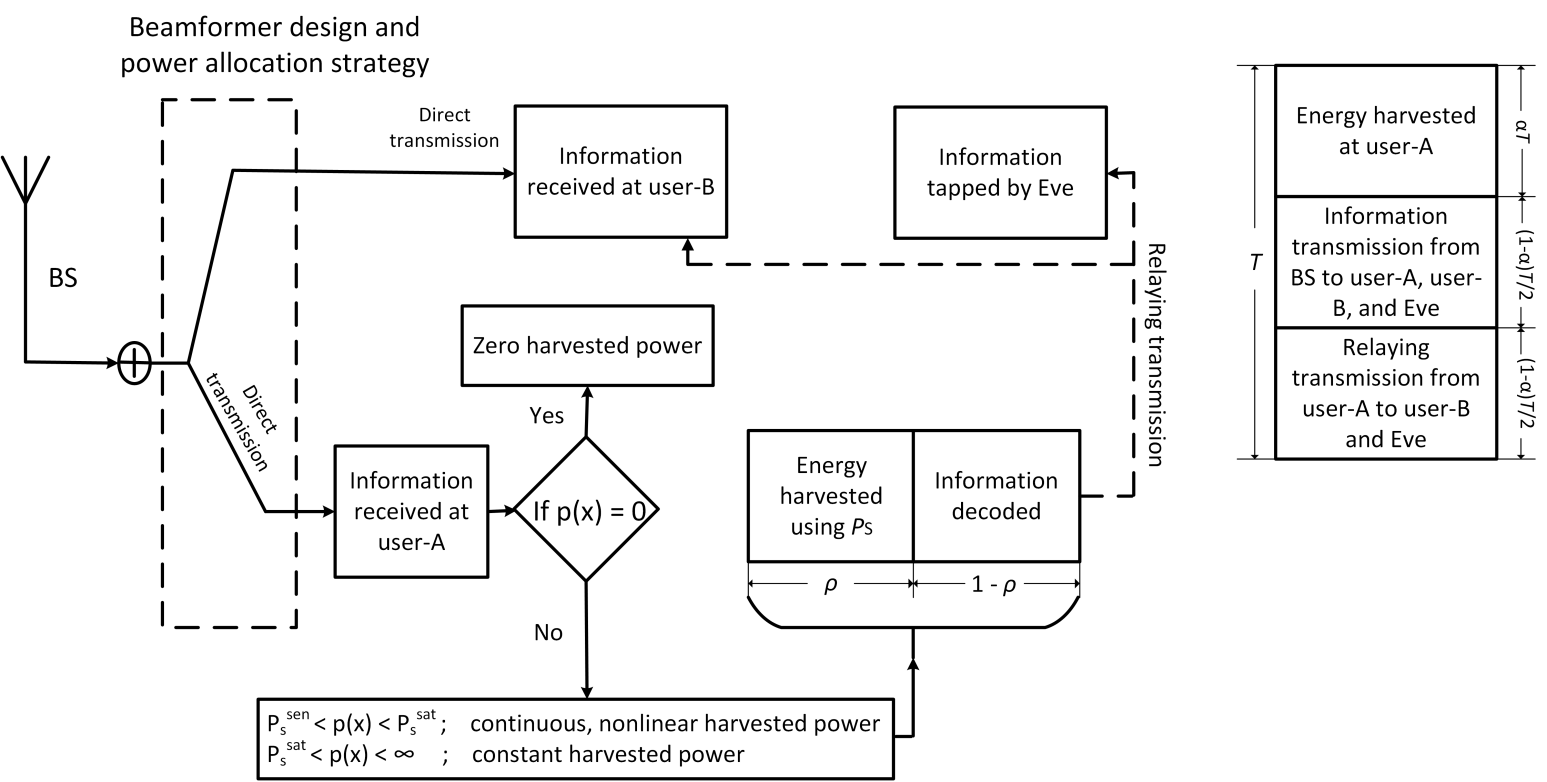
A hybrid simultaneous wireless information and power transmission (SWIPT) time switching/power splitting (TS/PS) protocol.

In the proposed model, user-A acts as a hybrid TS/PS relay where the time switching block *T* is split into three parts: In the first time block, user-A harvests the energy from the BS utilizing the time duration αT. In the second time block, i.e., $\left(1-\alpha \right)\frac{T}{2}$, power splitting takes place, where the user-A utilizes a power fraction ρ of the received power for energy harvesting and the remaining power fraction 1−ρ to decode information simultaneously. In the third time block having $\left(1-\alpha \right)\frac{T}{2}$ time duration the relaying transmission takes place where user-A utilizes its harvested energy to power the relaying operation. The ranges of power splitting and time switching parameters are 0<ρ<1 and 0<α<1, respectively. Presence of rectifier circuits in a system imposes nonlinearity in the power conversion. Furthermore, the harvesting power circuit shows zero response if the input power falls below a sensitivity threshold ($P_{S}^{\mathrm{sen}}$). If the input power is between the sensitivity and saturation threshold ($P_{S}^{\mathrm{sat}}$), the harvested power is a continuous, nonlinear, and increasing function of the input power. For the input power above saturation, the output power of the harvester is saturated as discussed in [35,36]. Therefore, the harvested power at the output of the harvesting circuit is

$$\begin{array}{cc} p(x)= & \left\{\begin{array}{c} 0,\;x\in [0,P_{S}^{\mathrm{sen}}],\\ E_{A}\left(x\right)\cdot x,\;x\in \left[P_{S}^{\mathrm{sen}},P_{S}^{\mathrm{sat}}\right],\\ E_{A}\left(P_{S}^{\mathrm{sat}}\right)\cdot P_{S}^{\mathrm{sat}},\;x\in \left[P_{S}^{\mathrm{sat}},\infty \right], \end{array}\right. \end{array}$$

where $E_{A}\left(\cdot \right)$ is the harvesting efficiency which is a function of input power. The hybrid SWIPT TS/PS protocol is shown in Figure 2. Here, we assume that Eve is assumed to be located close to the cell-edge. SINR is elaborated in the following stages.

### 2.1. First Time Slot

In the first time slot, information is transmitted from BS to user-A, user-B, and Eve. The message signals $s_{B}$ and $s_{A}$ of user-B and user-A, respectively, are superposed as $w\left(\sqrt{p_{A}}s_{A}+\sqrt{p_{B}}s_{B}\right)$ and then broadcast towards all the users through *N* number of transmit antennas of BS, where $p_{A}$ and $p_{B}$ are the power allocations coefficients. Furthermore, following the principle of NOMA, it is assumed that $p_{A}$ + $p_{B}$ = 1, and $0<p_{A}<p_{B}$. The information received during first time slot is divided as follows.

#### 2.1.1. From BS to User-A

The information received at user-A is written as

(1) $$\begin{array}{cc} y_{A} & =wh_{A}^{H}\left(\sqrt{p_{A}P_{S}}s_{A}+\sqrt{p_{B}P_{S}}s_{B}\right)+n_{A}\left(n\right), \end{array}$$

where the first term is the user-A’s desired message, the second term is the interference for user-A, and the remaining term is denoted as AWGN. Here, $h_{A}$ is the channel of order N×1 between BS to user-A.

Now, the energy harvested by user-A using hybrid SWIPT TS/PS protocol also takes place in the first time slot as follows:

(2) $$\begin{array}{cc} E_{A}= & \eta P_{s}|h_{A}^{H}{w|}^{2}\alpha T+\eta \rho P_{s}{\left|h_{A}^{H}w\right|}^{2}\left(1-\alpha \right)T/2\\ = & \left|\right|h_{A}{\left|\right|}_{ww^{H}\left(\eta P_{s}\alpha T+\eta \rho P_{s}\left(1-\alpha \right)T/2\right)}^{2}, \end{array}$$

where we achieve the second equality by quadratic formulation (The quadratic formulation for any matrix X is written as ${\left||h|\right|}_{X}^{2}≜h^{H}Xh$.), and the energy conversion efficiency denoted by η having the range 0<η<1 is used to decode the information.

Now, the SIC receiver is applied at user-A, and following the scheme of NOMA and superposition principle, user-A first decodes the message signal of user-B, i.e., $s_{B}$ and then detects its own message $s_{A}$ by subtracting the user-B’s decoded message.

Now, the SINR at user-A to decode the message signal of user-B is written as   

(3) $$\begin{array}{cc} \Gamma_{A}^{B}= & \frac{\left(1-\rho \right)p_{B}P_{s}{\left|h_{A}^{H}w\right|}^{2}}{\left(1-\rho \right)p_{A}P_{s}{\left|h_{A}^{H}w\right|}^{2}+\left(1-\rho \right)\sigma_{a}^{2}+\sigma_{c}^{2}}\\ = & \frac{\left|\right|h_{A}{\left|\right|}_{ww^{H}\left(\left(1-\rho \right)p_{B}P_{s}\right)}^{2}}{\left|\right|h_{A}{\left|\right|}_{ww^{H}\left(\left(1-\rho \right)p_{A}P_{s}\right)}^{2}+\left(1-\rho \right)\sigma_{a}^{2}+\sigma_{c}^{2}}\\ = & \frac{\left|\right|{\bar{h}}_{A}{\left|\right|}_{U}^{2}}{\left|\right|{\bar{h}}_{A}{\left|\right|}_{\tilde{U}}^{2}+\left(1-\rho \right)\sigma_{a}^{2}+\sigma_{c}^{2}}. \end{array}$$

In (3), by using the the quadratic formulation we achieve the second equality, and by means of whitened transformation, i.e., ${\bar{h}}_{A}$ from relation (5) in [37] we achieve the third equality. Furthermore, ${\bar{h}}_{A}=R_{A}^{-\frac{H}{2}}h_{A}$, where $h_{A}∼\mathrm{CN}\left(0,R_{A}\right)$$\Rightarrow {\bar{h}}_{N}∼\mathrm{CN}\left(0,I\right)$. Moreover, for simplification, $U=R_{A}^{\frac{1}{2}}ww^{H}R_{A}^{\frac{H}{2}}\left(1-\rho \right)p_{B}P_{s}$ and $\tilde{U}=R_{A}^{\frac{1}{2}}ww^{H}R_{A}^{\frac{H}{2}}\left(1-\rho \right)p_{A}P_{s}$ are considered as the weight matrices of user-B and user-A, respectively, while $\sigma_{a}^{2}$ and $\sigma_{c}^{2}$ are the variance terms at the receiving antennas.

Now, after subtracting the message signal of user-B from the composite signal, user-A can easily decode its own message without facing interference. Therefore, at user-A the received SNR to decode its own message $s_{A}$, denoted as $\Gamma_{A}^{A}$, can be expressed as

(4) $$\begin{array}{c} \Gamma_{A}^{A}=\frac{\left|\right|{\bar{h}}_{A}{\left|\right|}_{U}^{2}}{\left(1-\rho \right)\sigma_{a}^{2}+\sigma_{c}^{2}}. \end{array}$$

#### 2.1.2. From BS to User-B

During the direct transmission stage, the SNR at user-B is characterized given that the user-B has higher transmit power, and SIC is applied at user-A. Therefore, user-B deals the interference due to user-A as noise. Thus, the SNR at user-B to decode its own message is written as

(5) $$\begin{array}{cc} \Gamma_{B}^{B}= & \frac{p_{B}P_{s}{\left|h_{B}^{H}w\right|}^{2}}{p_{A}P_{s}{\left|h_{B}^{H}w\right|}^{2}+\sigma_{a}^{2}+\sigma_{c}^{2}}\\ = & \frac{\left|\right|{\bar{h}}_{B}{\left|\right|}_{V}^{2}}{\left|\right|{\bar{h}}_{B}{\left|\right|}_{\tilde{V}}^{2}+\sigma_{a}^{2}+\sigma_{c}^{2}}, \end{array}$$

where the channel in the second equality is achieved as ${\bar{h}}_{B}=R_{B}^{-\frac{H}{2}}h_{B}$, where $h_{B}∼\mathrm{CN}\left(0,R_{B}\right)$$\Rightarrow {\bar{h}}_{B}∼\mathrm{CN}\left(0,I\right)$. Furthermore, $V=R_{B}^{\frac{1}{2}}ww^{H}R_{B}^{\frac{H}{2}}\left(p_{B}P_{s}\right)$ is the desired weight matrix and $\tilde{V}=R_{B}^{\frac{1}{2}}ww^{H}R_{B}^{\frac{H}{2}}\left(p_{A}P_{s}\right)$ is the noise weight matrix for user-B.

#### 2.1.3. From BS to Eve

The information signal received at Eve is written as

(6) $$\begin{array}{cc} y_{E} & =wh_{E}^{H}\left(\sqrt{p_{A}P_{s}}s_{A}+\sqrt{p_{B}P_{s}}s_{B}\right)+n_{E}\left(n\right). \end{array}$$

The first term in (6) is the message signal of user-A which Eve taps from the BS, while the second term is the message signal of user-B that Eve can listen from the BS.

Now, we consider that Eve has the capability to differentiate message signals of both users. Therefore, the SINR for Eve to wiretap the information of user-A is expressed as

(7) $$\begin{array}{cc} \Gamma_{\mathrm{Eve}}^{A}= & \frac{p_{A}P_{s}{\left|h_{E}^{H}w\right|}^{2}}{p_{B}P_{s}{\left|h_{E}^{H}w\right|}^{2}+\sigma_{a}^{2}+\sigma_{c}^{2}}\\ = & \frac{\left|\right|{\bar{h}}_{E}{\left|\right|}_{E}^{2}}{\left|\right|{\bar{h}}_{E}{\left|\right|}_{\tilde{E}}^{2}+\sigma_{a}^{2}+\sigma_{c}^{2}}, \end{array}$$

where in the first equality $h_{E}$ is the channel link from BS to Eve. In the second equality, ${\bar{h}}_{E}$ is achieved by using the transformation ${\bar{h}}_{E}=R_{E}^{-\frac{H}{2}}h_{E}$. Moreover, the matrix $E=R_{E}^{\frac{1}{2}}ww^{H}R_{E}^{\frac{H}{2}}p_{A}P_{S}$, and $\tilde{E}=R_{E}^{\frac{1}{2}}ww^{H}R_{E}^{\frac{H}{2}}p_{B}P_{S}$ are the desired and noise weight matrices, respectively, for Eve.

Similarly, SINR for Eve to wiretap the information of user-B is written as

(8) $$\begin{array}{cc} \Gamma_{\mathrm{Eve}}^{B}= & \frac{\left|\right|{\bar{h}}_{E}{\left|\right|}_{\tilde{E}}^{2}}{\left|\right|{\bar{h}}_{E}{\left|\right|}_{E}^{2}+\sigma_{a}^{2}+\sigma_{c}^{2}}. \end{array}$$

### 2.2. Second Time Slot

The second time slot is for user-A to forward the information to user-B and Eve through cooperative relaying transmission. The information received from user-A at Eve and user-B is discussed in the following subsections.

#### 2.2.1. From User-A to Eve

Power provided for relaying process is accomplished in first time slot, as in [38]. In this time slot, the Eve has the capability to listen to message signal of user-B. The information received from user-A to Eve is written by using the expression (7) from in [39] as follows:

(9) $$\begin{array}{c} y_{\mathrm{AE}}=\sqrt{p_{A}}{\hat{s}}_{B}h_{\mathrm{AE}}+n_{a}+n_{c}, \end{array}$$

where ${\hat{s}}_{B}$ is the re-encoded version of $s_{B}$, and $p_{A}$ is the power of user-A harvested in the first time slot, and thus through this power, the cooperative relaying transmission takes place. The value of $p_{A}$ is obtained from the expression (7) of [39].

Now, the SINR received at Eve to listen the message signal of user-B through user-A to the Eve channel is written as

(10) $$\begin{array}{c} \Gamma_{\mathrm{AE}}^{B}=|h_{\mathrm{AE}}{|}^{2}\left|\right|h_{A}{\left|\right|}_{\left[\frac{ww^{H}\eta P_{s}\left(2\alpha +\left(1-\alpha \right)\rho \right)}{\left(\sigma_{a}^{2}+\sigma_{c}^{2}\right)\left(1-\alpha \right)}\right]}^{2}. \end{array}$$

The information at Eve is received in two stages; thus, both relayed and direct signals are combined at Eve. Therefore, considering the selection combining technique, the SINR at Eve to listen the information of user-B due to combination of these two signals is written as

(11) $$\begin{array}{c} \Gamma_{\mathrm{Eve}}=\max\left(\Gamma_{\mathrm{Eve}}^{B},\Gamma_{\mathrm{AB}}^{B}\right). \end{array}$$

#### 2.2.2. From User-A to User-B

Similarly, user-A also forwards the information toward user-B by using the energy which is harvested in the first time slot. Therefore, we write the SINR received at user-B through user-A to user-B channel as

(12) $$\begin{array}{c} \Gamma_{\mathrm{AB}}=|h_{\mathrm{AB}}{|}^{2}\left|\right|h_{A}{\left|\right|}_{\left[\frac{ww^{H}\eta P_{s}\left(2\alpha +\left(1-\alpha \right)\rho \right)}{\left(\sigma_{a}^{2}+\sigma_{c}^{2}\right)\left(1-\alpha \right)}\right]}^{2}. \end{array}$$

The information at user-B is also received in two stages. Consequently, both relayed and direct signals are combined at user-B. Thus, by using the selection combining technique again, the SINR at user-B due to combination of these two signals becomes

(13) $$\begin{array}{c} \Gamma_{B}=\max\left(\Gamma_{B}^{B},\Gamma_{\mathrm{AB}}\right). \end{array}$$

## 3. Outage Probability Characterization

The probability that a user’s instantaneous data rate drops below a predefined target SNR is known as the user’s outage probability. The outage probability performance of user-A, user-B, and Eve is investigated in this section for the MISO-NOMA system under Rayleigh fading channels. It is assumed that both the users are independent.

### 3.1. Outage Probability of User-B

The outage probability of user-B, denoted by $O_{B}$, by considering linearly dependent beamformers w is written as

(14) $$\begin{array}{cc} O_{B} & =Pr\left(\Gamma_{A}^{B}<\gamma_{\mathrm{th}},\Gamma_{B}^{B}<\gamma_{\mathrm{th}}\right)+Pr\left(\Gamma_{A}^{B}>\gamma_{\mathrm{th}},max\left(\Gamma_{B}^{B},\Gamma_{\mathrm{AB}}\right)<\gamma_{\mathrm{th}}\right). \end{array}$$

In (14), $\gamma_{\mathrm{th}}$ is the the SNR threshold to decode $s_{B}$, and is written as $\gamma_{\mathrm{th}}=2^{\frac{2R_{B}}{1-\alpha }}-1$, where $R_{B}$ is the data rate of user-B.

The closed-form expression for $O_{B}$ in terms of exponential function $\left(\exp(\cdot )\right)$, unit step function $\left(u(\cdot )\right)$, and generalized gamma function $\left(\Gamma (\cdot )\right)$, for linearly dependent beamformers is written as (15).

**Proof.**  The proof of (15) is given in Appendix A.    □

(15) $$\begin{array}{cc} O_{B}= & \left(1-\sum_{i=1}^{N}\frac{\lambda_{iB}}{\prod_{j=1,j\neq i}^{N}\left(\lambda_{iB}-\lambda_{jB}\right)}\exp\left(\frac{-\gamma_{\mathrm{th}}\left(\sigma_{a}^{2}+\sigma_{c}^{2}\right)}{\lambda_{iB}}\right)u\left(\frac{\gamma_{\mathrm{th}}\left(\sigma_{a}^{2}+\sigma_{c}^{2}\right)}{\lambda_{iB}}\right)\right)\left[\left(1-\sum_{i=1}^{N}\frac{\lambda_{iA}}{\prod_{j=1,j\neq i}^{N}\left(\lambda_{iA}-\lambda_{jA}\right)}\right.\right.\\ & \left.\left.\;\times \exp\left(\frac{-\gamma_{\mathrm{th}}\left(\left(1-\rho \right)\sigma_{a}^{2}+\sigma_{c}^{2}\right)}{\lambda_{iA}}\right)u\left(\frac{\gamma_{\mathrm{th}}\left(\left(1-\rho \right)\sigma_{a}^{2}+\sigma_{c}^{2}\right)}{\lambda_{iA}}\right)\right)+\left(\exp\left(-\frac{\gamma_{\mathrm{th}}}{\left(a_{1}-a_{2}\gamma_{\mathrm{th}}\right){.||w||}^{2}}\right)\right.\right.\\ & \left.\left.\;-\Gamma \left(1,\frac{\gamma_{\mathrm{th}}}{\left(a_{1}-a_{2}\gamma_{\mathrm{th}}\right){\left||w|\right|}^{2}};\frac{\gamma_{\mathrm{th}}}{{\left||w|\right|}^{2}l_{AB}.c}\right)\right)\right]. \end{array}$$

(16) $$\begin{array}{cc} O_{\mathrm{Eve}}= & \left(1-\sum_{i=1}^{N}\frac{\lambda_{iE}}{\prod_{j=1,j\neq i}^{N}\left(\lambda_{iE}-\lambda_{jE}\right)}\exp\left(\frac{-\gamma_{\mathrm{th}}\left(\sigma_{a}^{2}+\sigma_{c}^{2}\right)}{\lambda_{iE}}\right)u\left(\frac{\gamma_{\mathrm{th}}\left(\sigma_{a}^{2}+\sigma_{c}^{2}\right)}{\lambda_{iE}}\right)\right)\left[\left(1-\sum_{i=1}^{N}\frac{\lambda_{iA}}{\prod_{j=1,j\neq i}^{N}\left(\lambda_{iA}-\lambda_{jA}\right)}\right.\right.\\ & \left.\left.\;\times \exp\left(\frac{-\gamma_{\mathrm{th}}\left(\left(1-\rho \right)\sigma_{a}^{2}+\sigma_{c}^{2}\right)}{\lambda_{iA}}\right)u\left(\frac{\gamma_{\mathrm{th}}\left(\left(1-\rho \right)\sigma_{a}^{2}+\sigma_{c}^{2}\right)}{\lambda_{iA}}\right)\right)+\left(\exp\left(-\frac{\gamma_{\mathrm{th}}}{\left(a_{1}-a_{2}\gamma_{\mathrm{th}}\right){.||w||}^{2}}\right)\right.\right.\\ & \left.\left.\;-\Gamma \left(1,\frac{\gamma_{\mathrm{th}}}{\left(a_{1}-a_{2}\gamma_{\mathrm{th}}\right){\left||w|\right|}^{2}};\frac{\gamma_{\mathrm{th}}}{{\left||w|\right|}^{2}l_{AE}.c}\right)\right)\right]. \end{array}$$

(17) $$\begin{array}{cc} O_{A}= & 1-\sum_{i=1}^{N}\frac{\lambda_{iA}}{\prod_{j=1,j\neq i}^{N}\left(\lambda_{iA}-\lambda_{jA}\right)}\exp\left(\frac{-\gamma_{\mathrm{th}}\left(\left(1-\rho \right)\sigma_{a}^{2}+\sigma_{c}^{2}\right)}{\lambda_{iA}}\right)u\left(\frac{\gamma_{\mathrm{th}}\left(1-\rho \right)\sigma_{a}^{2}+\sigma_{c}^{2}}{\lambda_{iA}}\right). \end{array}$$

### 3.2. Outage Probability of Eve

The outage probability of Eve, denoted by $O_{\mathrm{Eve}}$, by considering the beamformers w to be linearly dependent, is written as

(18) $$\begin{array}{cc} O_{\mathrm{Eve}} & =Pr\left(\Gamma_{A}^{B}<\gamma_{\mathrm{th}},\Gamma_{\mathrm{Eve}}^{B}<\gamma_{\mathrm{th}}\right)\\ & +Pr\left(\Gamma_{A}^{B}>\gamma_{\mathrm{th}},max\left(\Gamma_{\mathrm{Eve}}^{B},\Gamma_{\mathrm{AE}}^{B}\right)<\gamma_{\mathrm{th}}\right). \end{array}$$

In (18), $\gamma_{\mathrm{th}}$ is the the SNR threshold to decode $s_{B}$, and is written as $\gamma_{\mathrm{th}}=2^{\frac{2R_{B}}{1-\alpha }}-1$, where $R_{B}$ is the data rate of user-B. It is assumed that both the users are independent.

The closed-form expression of $O_{Eve}$ can be written as (16), where the beamformers remain linearly dependent.

**Proof.**  The proof of (16) follows the same approach as given in Appendix A. Moreover, $\lambda_{iA}$ and $\lambda_{iB}$ are the eigenvalues of user-A and user-B obtained through their respective correlation matrices, while $l_{AE}$ is the distance from user-A to Eve.    □

### 3.3. Outage Probability of User-A

The outage probability of user-A, denoted by $O_{A}$, is written by considering the beamformers w to be linearly dependent, as 

(19) $$\begin{array}{cc} O_{A} & =Pr\left(\Gamma_{A}^{A}<\gamma_{\mathrm{th}}\right). \end{array}$$

In (19), $\gamma_{\mathrm{th}}$ is the the SNR threshold to detect $S_{A}$, and is written as $\gamma_{\mathrm{th}}=2^{\frac{2R_{A}}{1-\alpha }}-1$, where $R_{A}$ is the data rate of user-A. In the above equation, it is assumed that both the users are independent.

Now using (4) in (19), we get

(20) $$\begin{array}{cc} O_{A} & =Pr\left(\frac{\left|\right|{\bar{h}}_{A}{\left|\right|}_{U}^{2}}{\left(1-\rho \right)\sigma_{a}^{2}+\sigma_{c}^{2}}<\gamma_{\mathrm{th}}\right)\\ \; & =Pr\left(\left(\left(1-\rho \right)\sigma_{a}^{2}+\sigma_{c}^{2}\right)\gamma_{\mathrm{th}}-\left|\right|{\bar{h}}_{A}{\left|\right|}_{U}^{2}>0\right)\\ \; & =\int_{-\infty }^{\infty }p\left({\bar{h}}_{A}\right)u\left(\gamma_{\mathrm{th}}\left(\left(1-\rho \right)\sigma_{a}^{2}+\sigma_{c}^{2}\right)-\left|\right|{\bar{h}}_{A}{\left|\right|}_{U}^{2}\right)d{\bar{h}}_{A}. \end{array}$$

The exact closed-form expression of $O_{A}$ is written as (17).

**Proof.**  The proof of (17) follows the same approach as in the derivation of (22) in [40].    □

### 3.4. Secure Communication Criteria for User-B

In this subsection, we present secure communication criteria through an optimization problem. This work focuses on a scenario where user-A and user-B are trusted, which means that the SIC processing is perfectly done at user-A. Moreover, in our assumptions Eve is closer to user-B as compared to user-A or BS, which may lead to the degradation in outage probability specifically for user-B. Therefore, we propose a security enhancement algorithm through optimization problem which degrades the outage probability of Eve in such a way that we get lower outage probability of user-B, and also use the outage probability of user-A as a constraint to avoid the outage probability degradation of user-A. We select (15) as our objective function which is optimized with respect to beamformer vector w in the presence of constraints through which the transmit power is restricted. Thus, we write

(21) $$\begin{array}{cccc} \; & \underset{w}{\mathrm{minimize}}\; & \; & O_{B}\left(w\right)\\ \; & \mathrm{subject}\;\mathrm{to}\; & \; & {C1:||w||}^{2}=1\\ \; & \; & & C2:{\left(O_{A}\right)}^{i+1}\leq {\left(O_{A}\right)}^{i}\\ \; & \; & & C3:{\left(O_{\mathrm{Eve}}\right)}^{i+1}\geq {\left(O_{\mathrm{Eve}}\right)}^{i}, \end{array}$$

where the postscript *i* denotes the number of iterations. The constraint *C*1 indicates that $ww^{H}=1$, while the constraint *C*2 guarantees that $O_{A}$ can reach its minimum outage probability value, and the constraint *C*3 represents that the optimized outage probability of Eve should have higher value as compared to its initial value. The optimization problem given in (21) is a non-convex single objective constrained optimization problem, so we solve it through exhaustive search approach. A pseudocode for outage minimization problem in (21) is given in Algorithm 1.
**Algorithm 1** Pseudocode of optimized beamformer.**Input**: Initialized value of beamformer vector**Output**: Optimized beamformer value1.    Initialize w, set threshold level (ζ), precision, number of iteration, and time index (*i*)2.    Repeat3.      Compute $O_{B}$ as in (21)4.      i=i+15.      Compute ${\left(O_{B}\right)}^{i+1},{\left(O_{A}\right)}^{i+1}{\left(O_{\mathrm{Eve}}\right)}^{i+1}$ as in (21)6.      If $\left\{{\left(O_{A}\right)}^{i+1}\leq {\left(O_{A}\right)}^{i}\right\}$ and $\left\{{\left(O_{\mathrm{Eve}}\right)}^{i+1}\geq {\left(O_{\mathrm{Eve}}\right)}^{i}\right\}$7.       Store beamformer value, $O_{A}$, and $O_{\mathrm{Eve}}$ then perform recursion8.       Else9.       Return optimized beamformer value, $O_{A}$, and $O_{\mathrm{Eve}}$10.      Stop algorithm = true11.     End if12.   Until (Stop algorithm = true)

## 4. Numerical Results

In this section, numerical results are presented for the cooperative MISO-NOMA networks performance for the outage probability over Rayleigh fading channels. For simulation, we use MATLAB programming tools with appropriate parameters. Monte Carlo simulations are used to validate the analytical closed-form expression of outage probability. We consider that the BS, user-A, and user-B are all in a straight line. This assumption has been widely adopted in cooperative NOMA networks [39,41]. The reason behind this assumption is to discuss the effect of position of user-A on the performance of the two-hop cooperative NOMA systems, where user-A moves along BS to user-B link. Assuming that the distance from BS to user-B $\left(d_{SB}\right)$ is 10 m, the distance from user-A to the BS $\left(d_{SA}\right)$ is 3 m and the distance between user-A and user-B is $d_{AB}=d_{SB}-d_{SA}$. The distinct correlation matrices of transmitter and receiver are given as $R_{ij-Tx}^{\frac{1}{2}}=\chi_{1}^{\left|i-j\right|}$ and $R_{ij-Rx}^{\frac{1}{2}}=\chi_{2}^{\left|i-j\right|}$, respectively, where the correlation coefficients $\chi_{1}$ and $\chi_{2}$ are bounded between 0 and 1. Unless stated otherwise, the values of simulation parameters used are mentioned in Table 1, which are extensively used in other works on MISO-NOMA. These parameters are loosely adopted from in [38,39].

Let us quickly review how power allocation coefficients (PACs) have been calculated in the literature in order to pick suitable PACs for our plots. Evidently, in NOMA, there are different methods for determining the PACs. The authors of [41,42,43] randomly selected the power coefficients as long as the NOMA’s principle is met, i.e., $p_{1}\leq p_{2}$ and $p_{1}+p_{2}=1$. These PACs can also be calculated depending on the user’s QoS and target data rate, as in [44]. We calculate the PACs dynamically in this paper, depending on the target data rates of both user-A and user-B, as done in [38], and given as follows:

$$\begin{array}{c} \left\{\begin{array}{cc} p_{1}= & \frac{2^{2R_{th1}}-1}{2^{2R_{th1}+2R_{th2}}-1}\\ p_{2}= & 1-p_{1}. \end{array}\right. \end{array}$$

**Figure 3:**
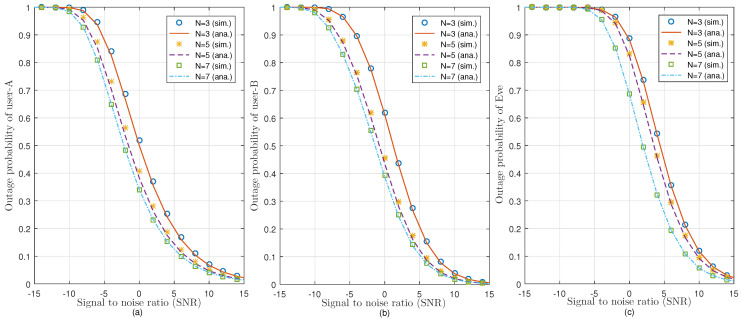
Effect of different values of BS transmit antennas on the outage probability of (**a**) user-A, (**b**) user-B, and (**c**) Eve, against SNR in dB.

Figure 3a–c presents the OP of user-A, user-B, and Eve as a function of SNR, respectively, for validation purposes. The results derived from closed-form OP expressions are well matched with the simulation results, which indicates the validation of our model. It can also be observed that user-A shows better outage probability among others, while the outage probability of undesired user, i.e., Eve has the worst outage probability. This is because the distance between BS and Eve is assumed as $d_{SE}=d_{SB}+5m$. Furthermore, the impact of antenna diversity can also be seen in Figure 3. By increasing the number of antennas at the transmitter side, it results in better outage probability. Similarly, it can also be observed that the outage probability of the users gets improved at higher values of SNR.

**Figure 4:**
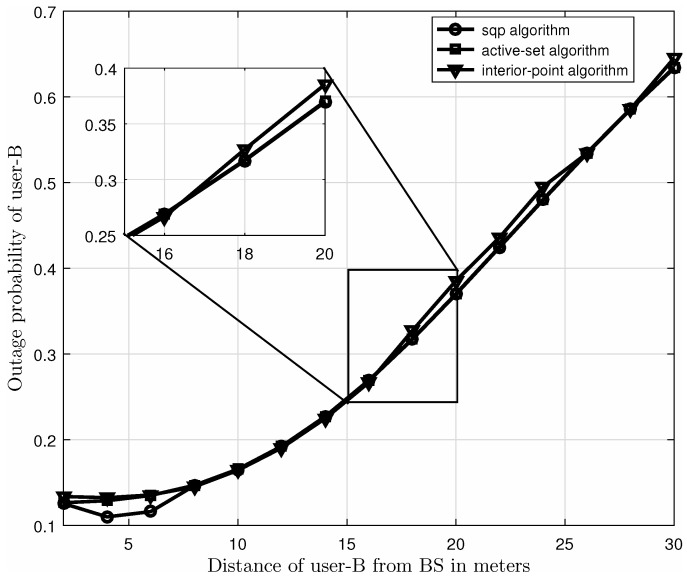
Outage probability comparison of “sqp”, “active-set”, and “interior-point” algorithms for specification of experiment.

Now, in Figure 4 we explain the complexity of our model by computing the execution time of the algorithm. The execution times in seconds, of sequential quadratic programming (“sqp”), “active-set”, and “interior-point” algorithms are 1.250742 s, 0.278754 s, and 2.700135 s, respectively. Intel${}^{®}$ Core${}^{\mathrm{TM}}$ i7-7500U CPU @ 2.70 GHz 2.90 GHz is used with 8GB RAM. Furthermore, the outage probability comparison of these three algorithms is shown in Figure 4, where the “sqp” algorithm gives lower outage probability as compared to “active-set” and “interior-point” algorithms specially for smaller distance of user-B from BS. For the rest of the experiments, we have considered “sqp” as optimization algorithm due to its superior performance.

**Figure 5:**
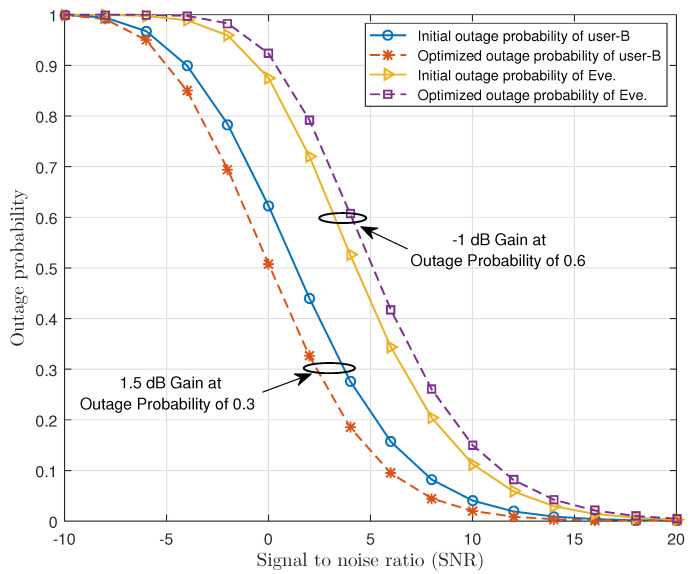
Comparison of outage probability of user-B and Eve before and after optimization versus SNR in dB.

Figure 5 presents the comparison of outage probability before and after the optimization of user-B and Eve. The positive gain of 1.5 dB, achieved by the user-B after the optimization, can be observed in this figure. Note that without degrading the outage probability of user-A, we have improved the optimized outage probability of user-B by 12%. At the same time, degradation in the outage probability of Eve by 10% and the negative gain of 1 dB is also achieved. This is because we have adjusted the beam from BS to user-B by using beamformer vector w in such a way that the beam becomes narrowed and is directed towards the legitimate user so that it becomes difficult for Eve to access the message signal of user-B. Similarly, Figure 5 can also be observed with respect to SNR, i.e., higher values of SNR result in better outage probability of the users.

**Figure 6:**
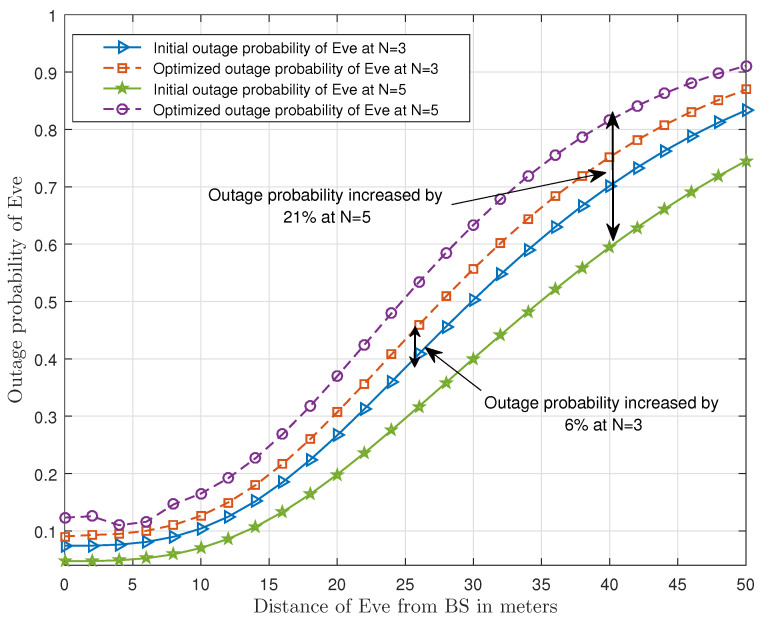
Comparison of outage probability of Eve with the distance of Eve from the BS.

Next, the distance of Eve from the BS is studied more deeply in Figure 6. It can be observed that going away from the BS degrades the performance of Eve. The difference between the initial and optimized outage probability values of Eve can also be seen in Figure 6. It can be observed that the optimized outage probability of Eve is increased by 6% and 21% when the number of transmit antennas are N=3 and N=5, respectively. Additionally, an important result, which we have depicted in Figure 6, is that we achieve the higher gain value by increasing the number of transmit antennas. A reason herein is that the Eve is far from the BS, thus the channel conditions between BS and Eve are poor.

**Figure 7:**
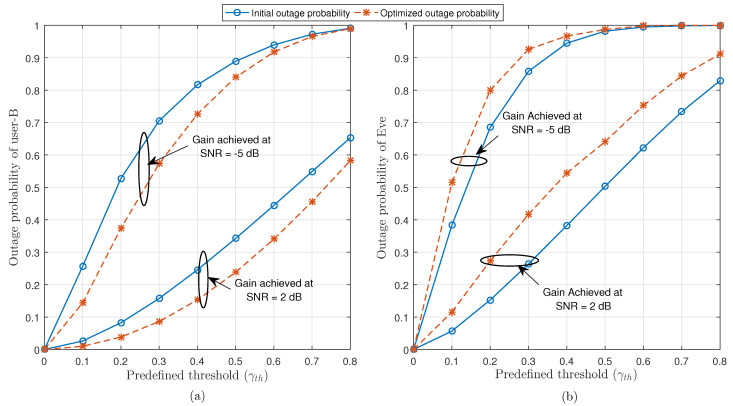
Impact of predefined threshold $\left(\gamma_{th}\right)$ at N=7 on the outage probability of (**a**) user-B and (**b**) Eve.

Figure 7 indicates the behaviour of outage probability of user-B and Eve under different values of predefined threshold $\gamma_{th}$. It is observed that the proposed optimal beamforming got significant gain at two different values of SNR in case of both user-B and Eve as shown in Figure 7a,b, respectively. Note that at different values of SNR, the optimized outage probability of user-B performs better as compared to its initial outage probability (before optimization). The optimized outage probability of Eve is degraded as compared to its initial outage probability by applying the proposed optimal beamforming algorithm, which ensures the accomplishment of our goal about secure communication for cell-edge user.

**Figure 8:**
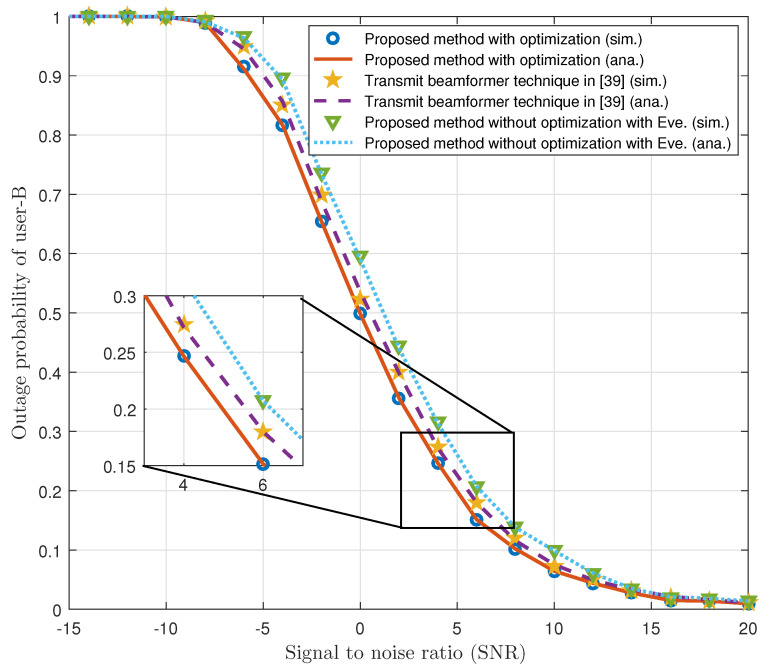
Outage probability comparison between transmit beamformer technique and our proposed model.

Last, Figure 8 presents a comparison of our proposed model with transmit beamformer-based technique [39] for the analysis of the outage probability of user-B in a similar network configuration. The main difference between the proposed work and the work in [39] is the general channel model. The physical layer security in MISO-NOMA system in the presence of an eavesdropper and secure communication criteria for the legitimate users is discussed in our work, which was not addressed in [39]. It is observed in Figure 8 that the proposed method with optimization performs better as compared to the work in [39]. However, without optimization, the proposed method shows higher outage probability of user-B as compared to the technique in [39]. This is due to the presence of Eve, and although Eve is also present in case of optimization in the proposed model, our optimization algorithm degrades the outage probability of Eve and improves the outage probability of user-B.

## 5. Conclusions

In this paper, we optimized the beamforming vector in downlink MISO NOMA systems in order to achieve lowest outage probability of cell-edge user. Specifically, we first provided the exact closed-form expression of outage probability of near-user, far-user, and eavesdropper under the parameters including the power allocation coefficients, transmit correlation, time switching/power splitting factors, and transmit antenna diversity. In addition, an optimization problem was developed for the case of two users, and an eavesdropper, which was then solved via the exhaustive search method. A secure MISO-NOMA communication is achieved by obtaining the best minimum outage probability of cell-edge user and performance degradation of an eavesdropper. Analytical results are validated by Monte Carlo simulation results, and antenna diversity at the transmitter is used to make significant performance gains. This work can be further expanded for multiple near-user MISO-NOMA systems acting as relays and by performing optimal analysis on power allocation coefficients to pick a best near-user to enhance overall performance of the system. Furthermore, the secrecy performance analysis of a MIMO-NOMA and massive MIMO-NOMA network in the presence of a multiple-antenna eavesdropper can be useful extensions of this work.

## Figures and Tables

**Table 1:** Simulation parameters.

Parameters	Values
Bandwidth	1 MHz
Correlation coefficient $\chi_{1}$	0.2+0.4j
Correlation coefficient $\chi_{2}$	0.1+0.3j
Data rates of users, $R_{th1}$ = $R_{th2}$	0.17 bits/s/Hz
Energy conversion efficiency to decode information, η	0.7
Noise power density of antenna, $n_{a}$	−100 dBm/Hz
Noise density to process information, $n_{c}$	−90 dBm/Hz
Number of Monte Carlo simulation runs	$10^{6}$
Path loss exponent, ϵ	3
The distance from BS to user-A, $d_{\mathrm{SA}}$	3 m
The distance from BS to user-B, $d_{\mathrm{SB}}$	10 m
The distance from user-A to user-B, $d_{\mathrm{AB}}$	$d_{\mathrm{SB}}-d_{\mathrm{SA}}$
The distance from BS to Eve, $d_{\mathrm{SE}}$	$d_{\mathrm{SB}}+5m$

## Abbreviations

The following abbreviations are used in this manuscript:

α	Time fraction
ρ	Power fraction
w	Beamformer vector
${\left||h|\right|}^{2}$	Norm-2 of vector h
$R_{ij-Tx}$	Transmit correlation matrix
$R_{ij-Rx}$	Receive correlation matrix
$P_{S}$	Transmitted Power
*N*	Base station antennas
$O_{\mathrm{Eve}}$	Outage Probability of Eve
$O_{A}$	Outage Probability of user-A
$O_{B}$	Outage Probability of user-B

## Appendix A

The beamformer vectors are linearly dependent, so (14) can be written as

(A1) $$\begin{array}{ccc} O_{B} & =Pr\left(\Gamma_{A}^{B}<\gamma_{th}\right)Pr\left(\Gamma_{B}^{B}<\gamma_{th}\right)+Pr\left(\Gamma_{B}^{B}<\gamma_{th}\right)Pr\left(\Gamma_{A}^{B}>\gamma_{th},\Gamma_{\mathrm{AB}}<\gamma_{th}\right) & \\ \; & =Pr\left(\Gamma_{B}^{B}<\gamma_{th}\right)\left[Pr\left(\Gamma_{A}^{B}<\gamma_{th}\right)+Pr\left(\Gamma_{A}^{B}>\gamma_{th},\Gamma_{\mathrm{AB}}<\gamma_{th}\right)\right]. \end{array}$$

Now, plugging (3), (5), and (12) in (A1), we get

(A2) $$\begin{array}{cc} O_{B} & =\underset{P_{1}}{\underset{︸}{Pr\left(\frac{\left|\right|{\bar{h}}_{A}{\left|\right|}_{U}^{2}}{\left|\right|{\bar{h}}_{A}{\left|\right|}_{\tilde{U}}^{2}+\left(1-\rho \right)\sigma_{a}^{2}+\sigma_{c}^{2}}<\gamma_{th}\right)}}\left[\underset{P_{2}}{\underset{︸}{Pr\left(\frac{\left|\right|{\bar{h}}_{B}{\left|\right|}_{V}^{2}}{\left|\right|{\bar{h}}_{B}{\left|\right|}_{\tilde{V}}^{2}+\sigma_{a}^{2}+\sigma_{c}^{2}}<\gamma_{th}\right)}}\right.\\ & \left.\;+\underset{P_{3}}{\underset{︸}{Pr\left(\Gamma_{A}^{B}>\gamma_{th},\Gamma_{\mathrm{AB}}<\gamma_{th}\right)}}\right], \end{array}$$

where linearly dependent beamformers are as a result written in form of matrices U, $\tilde{U}$, V, and $\tilde{V}$. Now, to solve $P_{1}$, we utilize quadratic formulation and u(x), where u(x) is the unit step function, i.e., $u\left(x\right)=\frac{1}{2\pi }\int_{-\infty }^{\infty }\frac{e^{x(j\omega +\beta )}}{j\omega +\beta }d\omega$; β>0. The presence of u(x) helps in the tractability of analysis in this work. Therefore, the closed-form expression of $P_{1}$ is written, by using (13) from in [39], as

(A3) $$\begin{array}{cc} P_{1} & =\left(1-\sum_{i=1}^{N}\frac{\lambda_{iB}}{\prod_{j=1,j\neq i}^{N}\left(\lambda_{iB}-\lambda_{jB}\right)}\exp\left(\frac{-\gamma_{th}\left(\sigma_{a}^{2}+\sigma_{c}^{2}\right)}{\lambda_{iB}}\right)u\left(\frac{\gamma_{\mathrm{th}}\left(\sigma_{a}^{2}+\sigma_{c}^{2}\right)}{\lambda_{iB}}\right)\right), \end{array}$$

Now, to solve $P_{2}$, same approach is opted as (A3) and written as follows:

(A4) $$\begin{array}{cc} P_{2} & =\left(1-\sum_{i=1}^{N}\frac{\lambda_{iA}}{\prod_{j=1,j\neq i}^{N}\left(\lambda_{iA}-\lambda_{jA}\right)}\times \exp\left(\frac{-\gamma_{th}\left(\left(1-\rho \right)\sigma_{a}^{2}+\sigma_{c}^{2}\right)}{\lambda_{iA}}\right)\right.\\ & \left.\;\times u\left(\frac{\gamma_{th}\left(\left(1-\rho \right)\sigma_{a}^{2}+\sigma_{c}^{2}\right)}{\lambda_{iA}}\right)\right). \end{array}$$

Next, to solve $P_{3}$, the link between user-A and user-B follows the exponential distribution. By utilizing the change in variable approach as in (40) from in [39] and generalized incomplete gamma function [45], the exact closed-form expression of $P_{3}$ is written as

(A5) $$\begin{array}{cc} P_{3} & =\Gamma \left(1,\frac{\gamma_{\mathrm{th}}}{\left(a_{1}-a_{2}\gamma_{th}\right){\left||w|\right|}^{2}};\frac{\gamma_{\mathrm{th}}}{{\left||w|\right|}^{2}l_{AB}.c}\right). \end{array}$$

In (A5), $a_{1}=\frac{\left(1-\rho \right)p_{B}P_{s}}{\left(1-\rho \right)\sigma_{a}^{2}+\sigma_{c}^{2}}$, $a_{2}=\frac{\left(1-\rho \right)p_{A}P_{s}}{\left(1-\rho \right)\sigma_{a}^{2}+\sigma_{c}^{2}}$, and $c=\frac{\left(\eta P_{s}\right)\left(\frac{2\alpha }{1-\alpha }+\rho \right)}{\sigma_{a}^{2}+\sigma_{c}^{2}}$, while $l_{AB}$ is the distance between user-A to user-B. Moreover, $\lambda_{iA}$ and $\lambda_{iB}$ are the eigenvalues of user-A and user-B obtained through their respective correlation matrices.

Finally, using (A3)–(A5) in (A2), we obtain the exact closed-form expression written as (15).

## References

[1] Al-Abbasi Z.Q., Khamis M.A. (2021). Spectral efficiency (SE) enhancement of NOMA system through iterative power assignment. Wirel. Netw..

[2] Cao K., Wang B., Ding H., Lv L., Tian J., Hu H., Gong F. (2021). Achieving Reliable and Secure Communications in Wireless-Powered NOMA Systems. IEEE Trans. Veh. Technol..

[3] Dai L., Wang B., Ding Z., Wang Z., Chen S., Hanzo L. (2018). A survey of non-orthogonal multiple access for 5G. IEEE Commun. Surv. Tutor..

[4] Dai X., Zhang Z., Bai B., Chen S., Sun S. (2018). Pattern division multiple access: A new multiple access technology for 5G. IEEE Wirel. Commun..

[5] Ding Z., Schober R., Poor H.V. (2020). Unveiling the Importance of SIC in NOMA Systems Part 1: State of the Art and Recent Findings. IEEE Commun. Lett..

[6] Lv L., Chen J., Ni Q., Ding Z., Jiang H. (2018). Cognitive non-orthogonal multiple access with cooperative relaying: A new wireless frontier for 5G spectrum sharing. IEEE Commun. Mag..

[7] Chen Z., Ding Z., Dai X., Zhang R. (2017). An optimization perspective of the superiority of NOMA compared to conventional OMA. IEEE Trans. Signal Process..

[8] Ding Z., Peng M., Poor H.V. (2015). Cooperative non-orthogonal multiple access in 5G systems. IEEE Commun. Lett..

[9] Zhai D., Zhang R., Cai L., Li B., Jiang Y. (2018). Energy-efficient user scheduling and power allocation for NOMA-based wireless networks with massive IoT devices. IEEE Internet Things J..

[10] Zaidi S.K., Hasan S.F., Gui X. (2020). Two-way SWIPT-aided hybrid NOMA relaying for out-of-coverage devices. Wirel. Netw..

[11] Gong J., Chen X. (2017). Achievable rate region of non-orthogonal multiple access systems with wireless powered decoder. IEEE J. Sel. Areas Commun..

[12] Wang J., Song X., Ma Y., Xie Z. (2020). Power Efficient Secure Full-Duplex SWIPT Using NOMA and D2D with Imperfect CSI. Sensors.

[13] Rajaram A., Khan R., Tharranetharan S., Jayakody D.N.K., Dinis R., Panic S. (2019). Novel SWIPT schemes for 5G wireless networks. Sensors.

[14] Boshkovska E., Ng D.W.K., Zlatanov N., Schober R. (2015). Practical non-linear energy harvesting model and resource allocation for SWIPT systems. IEEE Commun. Lett..

[15] Wang Y., Wu Y., Zhou F., Chu Z., Wu Y., Yuan F. (2017). Multi-objective resource allocation in a NOMA cognitive radio network with a practical non-linear energy harvesting model. IEEE Access.

[16] Huang Y., Li Z., Zhou F., Zhu R. (2017). Robust AN-aided beamforming design for secure MISO cognitive radio based on a practical nonlinear EH model. IEEE Access.

[17] Darsena D., Gelli G., Verde F. (2019). Cloud-aided cognitive ambient backscatter wireless sensor networks. IEEE Access.

[18] Tuan V.P., Hong I.P. (2020). Secure Communication in Cooperative SWIPT NOMA Systems with Non-Linear Energy Harvesting and Friendly Jamming. Sensors.

[19] Li X., Huang M., Zhang C., Deng D., Rabie K.M., Ding Y., Du J. (2019). Security and reliability performance analysis of cooperative multi-relay systems with nonlinear energy harvesters and hardware impairments. IEEE Access.

[20] Zhou Z., Chen X., Zhang Y., Mumtaz S. (2020). Blockchain-empowered secure spectrum sharing for 5G heterogeneous networks. IEEE Netw..

[21] Zhang C., Jia F., Zhang Z., Ge J., Gong F. (2020). Physical layer security designs for 5G NOMA systems with a stronger near-end internal eavesdropper. IEEE Trans. Veh. Technol..

[22] Melki R., Noura H.N., Chehab A. (2020). Physical layer security for NOMA: Limitations, issues, and recommendations. Ann. Telecommun..

[23] Madeira J., Guerreiro J., Serra H., Dinis R., Montezuma P., Campos L.M. (2020). A Physical Layer Security Technique for NOMA Systems with MIMO SC-FDE Schemes. Electronics.

[24] Ahiadormey R.K., Anokye P., Jo H.S., Song C., Lee K.J. (2021). Secrecy Outage Analysis in NOMA Power Line Communications. IEEE Commun. Lett..

[25] Zaghdoud N., Alouane W.H., Boujemaa H., Touati F. Secure performance of AF and DF relaying in cooperative NOMA systems. Proceedings of the 2019 19th International Conference on Sciences and Techniques of Automatic Control and Computer Engineering (STA).

[26] Yuan C., Tao X., Li N., Ni W., Liu R.P., Zhang P. (2019). Analysis on secrecy capacity of cooperative non-orthogonal multiple access with proactive jamming. IEEE Trans. Veh. Technol..

[27] Li B., Qi X., Huang K., Fei Z., Zhou F., Hu R.Q. (2018). Security-reliability tradeoff analysis for cooperative NOMA in cognitive radio networks. IEEE Trans. Commun..

[28] Lei H., Yang Z., Park K.H., Ansari I.S., Guo Y., Pan G., Alouini M.S. (2018). Secrecy outage analysis for cooperative NOMA systems with relay selection scheme. arXiv.

[29] Feng Y., Yan S., Liu C., Yang Z., Yang N. (2018). Two-stage relay selection for enhancing physical layer security in non-orthogonal multiple access. IEEE Trans. Inf. Forensics Secur..

[30] Liu Y., Qin Z., Elkashlan M., Gao Y., Hanzo L. (2017). Enhancing the physical layer security of non-orthogonal multiple access in large-scale networks. IEEE Trans. Wirel. Commun..

[31] Zeng M., Nguyen N.P., Dobre O.A., Poor H.V. (2019). Securing downlink massive MIMO-NOMA networks with artificial noise. IEEE J. Sel. Top. Signal Process..

[32] Nguyen N.P., Dobre O.A., Nguyen L.D., Nguyen C.T., Poor H.V. Secure downlink massive MIMO NOMA network in the presence of a multiple-antenna eavesdropper. Proceedings of the ICC 2019-2019 IEEE International Conference on Communications (ICC).

[33] Yue X., Liu Y., Yao Y., Li X., Liu R., Nallanathan A. (2020). Secure communications in a unified non-orthogonal multiple access framework. IEEE Trans. Wirel. Commun..

[34] Gomez G., Martin-Vega F.J., Lopez-Martinez F.J., Liu Y., Elkashlan M. (2019). Physical layer security in uplink NOMA multi-antenna systems with randomly distributed eavesdroppers. IEEE Access.

[35] Alevizos P.N., Bletsas A. (2018). Sensitive and nonlinear far-field RF energy harvesting in wireless communications. IEEE Trans. Wirel. Commun..

[36] Wang S., Xia M., Huang K., Wu Y.C. (2017). Wirelessly powered two-way communication with nonlinear energy harvesting model: Rate regions under fixed and mobile relay. IEEE Trans. Wirel. Commun..

[37] Hassan A.K., Moinuddin M., Al-Saggaf U.M., Aldayel O., Davidson T.N., Al-Naffouri T.Y. (2020). Performance Analysis and Joint Statistical Beamformer Design for Multi-User MIMO Systems. IEEE Commun. Lett..

[38] Do T.N., da Costa D.B., Duong T.Q., An B. (2017). Improving the performance of cell-edge users in MISO-NOMA systems using TAS and SWIPT-based cooperative transmissions. IEEE Trans. Green Commun. Netw..

[39] Ghous M., Abbas Z.H., Abbas G., Hassan A.K., Moinuddin M. (2020). Transmit beamformer based performance analysis and diversity gains of cell edge user in cooperative MISO-NOMA system. Phys. Commun..

[40] Hassan A.K., Moinuddin M., Al-Saggaf U.M., Al-Naffouri T.Y. (2017). Performance analysis of beamforming in MU-MIMO systems for Rayleigh fading channels. IEEE Access.

[41] Ding Z., Adachi F., Poor H.V. (2015). The application of MIMO to non-orthogonal multiple access. IEEE Trans. Wirel. Commun..

[42] Do N.T., Da Costa D.B., Duong T.Q., An B. (2016). A BNBF user selection scheme for NOMA-based cooperative relaying systems with SWIPT. IEEE Commun. Lett..

[43] Kim J.B., Lee I.H. (2015). Capacity analysis of cooperative relaying systems using non-orthogonal multiple access. IEEE Commun. Lett..

[44] Yang Z., Ding Z., Fan P., Al-Dhahir N. (2017). The impact of power allocation on cooperative non-orthogonal multiple access networks with SWIPT. IEEE Trans. Wirel. Commun..

[45] Chaudhry M.A., Zubair S.M. (2001). On a Class of Incomplete Gamma Functions with Applications.

